# Longterm Improvement After Cessation of Chronic Deep Brain Stimulation in Acquired Dystonia

**DOI:** 10.5334/tohm.620

**Published:** 2021-07-19

**Authors:** Marc E. Wolf, Christian Blahak, Christoph Schrader, Joachim K. Krauss

**Affiliations:** 1 Department of Neurology, Neurozentrum, Klinikum Stuttgart, Stuttgart, Germany; 2 Department of Neurology, Universitätsmedizin Mannheim, University of Heidelberg, Mannheim, Germany; 3 Department of Neurology, Ortenau Klinikum Lahr-Ettenheim, Lahr, Germany; 4 Department of Neurology, Hannover Medical School, Germany; 5 Department of Neurosurgery, Hannover Medical School, Germany

**Keywords:** deep brain stimulation, dystonia, battery depletion, globus pallidus internus, ventral intermediate nucleus

## Abstract

**Introduction:**

Deep brain stimulation (DBS) has become an accepted treatment for inherited and idiopathic dystonia but less so for acquired dystonia. Patients benefit from long-term improvement with chronic DBS. Prolonged benefit over months has even been reported after cessation of stimulation on long-term follow-up.

**Case report:**

We report a case of a 25-year-old man with acquired dystonia who had sustained symptom improvement despite battery depletion after 6.5 years of chronic bilateral thalamic and pallidal DBS.

**Discussion:**

We posit that chronic pallidal DBS can be a genuine disease-modifying treatment in single patients with dystonia with regard to its long-term effect even after prolonged discontinuation.

**Highlights:**

Chronic deep brain stimulation (DBS) is an approved treatment for idiopathic and inherited dystonia. During the early course of chronic stimulation, cessation of DBS due to battery depletion results in rapid worsening of symptoms and rapid battery replacement is required. Few reports of sustained symptom relief in idiopathic dystonia have been published. We report a case of sustained symptom relief in acquired dystonia after DBS cessation which likely reflects neuroplasticity changes with a disease-modifying impact.

## Introduction

Deep brain stimulation (DBS) has become an accepted treatment for inherited and idiopathic dystonia but less so for acquired dystonia. There is growing evidence that the clinical benefit of DBS in dystonia can be sustained for 10 years or longer and that the long-term outcome may surpass short-term benefit. When stimulation is discontinued in the first years after stimulation, dystonic symptoms may recur rapidly [1]. A sustained effect, however, may be observed when stimulation is switched off after long term stimulation. While this phenomenon has been noted in patients with inherited or idiopathic dystonia [2,3,4], it has not received attention before in acquired generalized dystonia.

## Case report

This 25-year-old man (early follow-up published elsewhere) [5] suffered from generalized acquired dystonia with choreoathetotic movements due to perinatal asphyxia. Upon clinical examination, he also had an action-induced tremor with myoclonic jerks, mild spastic tetraparesis and ataxic gait. He was wheelchair-bound for longer distances and was not able to drink or eat by himself independently. Furthermore, he had severe dysarthria. The Burke-Fahn-Marsden (BFM) motor score was 80.5 (assessing axial and more peripheral dystonic symptoms), disability score 19 (describing dystonia-related problems in daily life). The patient was under no specific medication.

He underwent implantation of quadripolar DBS electrodes (Model 3387; Medtronic Inc., Minneapolis) bilaterally in the ventral intermediate nucleus (Vim) of the thalamus and bilaterally in the posteroventral lateral globus pallidus internus (GPi) guided by CT-stereotactic surgery and microelectrode recording. Postoperative stereotactic CT demonstrated appropriate placement of all four electrodes. After test stimulation an implantable pulse generator (IPG) (Kinetra; Medtronic Inc., Minneapolis) was implanted and connected to the thalamic DBS electrodes. Postoperatively, he had improvement of tremor and dystonia, he was able to grip objects and was more independent (BFM motor score 67.5; disability score 15 at 17 months postoperatively). Stimulation settings and the further course are shown in ***Table 1***.

**Table 1:** Time course of stimulation settings and adaptations according to the clinical evolution in a patient with DBS of the Vim and GPi for acquired dystonia and sustained clinical benefit after DBS switch-off.


MONTHS	TARGET/MODE	INTENSITY	FREQUENCY	PULSE WIDTH [µS]	CLINICAL OBSERVATION

POST-OP	ACTIVE CONTACTS	[V]	[HZ]		

Pre-OP	NA	NA	NA	NA	**BFM motor score 80.5 BFM disability score 19**

Post-OP	Vim/bipolar	L 3,0; R 3,0	130 Hz	210 µs	improved fine motor skills and dysarthria

	(1–/2+; 5–/6+)				

2 months	Vim/bipolar	L 3,2; R 3,2	130 Hz	210 µs	stable

	(1–/2+; 5–/6+)				

8 months	Vim/bipolar	L 3,4; R 3,4	130 Hz	210 µs	improved gait (dystonic posture of legs) and dysarthria

	(1–/2+; 5–/6+)				

17 months	Vim/bipolar	L 3,7; R 3,8	130 Hz	210 µs	**BFM motor score 67.5**
	
	(1–/2+; 5–/6+)				**BFM disability score 15**

30 months	Vim/bipolar	L 4,0; R 4,1	130 Hz	210 µs	stable

	(1–/2+; 5–/6+)				

38 months	**No stimulation**	–	–	–	fine motor skills 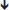 gait 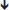 **BFM motor score 71,5** **BFM disability score 15**

41 months	GPi/bipolar	L 4,0; R 4,0	130 Hz	210 µs	improved fine motor skills (e.g. smartphone use)

	(1–/2+; 5–/6+)				

43 months	GPi/bipolar	L 4,3; R 4,3	130 Hz	210 µs	stable **BFM motor score 67** **BFM disability score 15**

	(1–/2+; 5–/6+)				

50 months	GPi/bipolar	L 4,7; R 3,9	130 Hz	210 µs	mild hypokinesia
	
	(1–/2+; 5–/6+)				gait 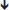

58 months	GPi/bipolar	L 4,5; R 3,9	130 Hz	210 µs	stable
	
	(1–/2+; 9–/10+)				IPG change

64 months	GPi/bipolar	L 4,5; R 3,8	130 Hz	210 µs	improved fine motor skills **BFM motor score 67** **BFM disability score 15**

	(1–/2+; 9–/10+)				

76 months	**No stimulation**	–	–	–	**BFM motor score 67,5**

					**BFM disability score 15**

105 months	**No stimulation**	–	–	–	**BFM motor score 58,5** **BFM disability score 13**


After 38 months of Vim stimulation the IPG was replaced upon battery depletion, and the previously and simultaneously implanted pallidal electrodes were then connected to possibly obtain further benefit. The rationale were the phasic and dystonic symptoms of the extremities, especially with severe difficulties to put the feet with the sole on the ground, and cervical dystonia further affecting the ability to eat. While there was subjective improvement in fine motor skills (e.g. handling of his smartphone became somewhat easier), the BFM motor and disability scores remained unchanged (BFM motor score 67, disability score 15) compared to bilateral Vim stimulation (electrodes were disconnected but not removed).

When he was seen for a routine follow-up 76 months after implantation, there was stable improvement of dystonia. However, IPG check revealed battery depletion since 5 weeks ago. Since clinical symptoms were stable with still an improvement of about 15% in the BFM motor score compared to the preoperative situation, IPG replacement was postponed and he continued to do well. Two and a half years after battery depletion, both the BFM motor score (58,5) and the disability score (13) were even further improved. No confounding factors such as medication, life circumstances or additional treatments had been modified. In the further course, however, one and a half year later, he noted a mild deterioration of dystonia and the IPG was replaced, leading to a rapid improvement to the former level, which is now stable for more than a year.

## Discussion

The mechanisms of chronic DBS in dystonia have not been fully clarified thus far. It appears that DBS does not only alter neuronal activity at the site of stimulation, but that it also has more widespread effects both on altered inhibition and neuroplasticity with a differing temporal profile [6,7]. Furthermore, it may alter more specifically network activity as shown by its impact on local field potentials [8,9].

Long-term efficacy of chronic DBS after several years has been shown in inherited and idiopathic dystonia for up to 10 years. The re-occurrence of dystonic symptoms after switching off of long-term chronic DBS has prospectively been studied in DYT 1 patients with GPi-DBS [6]. Patients had been stimulated for at least 5 years. Dystonic symptoms did not reach the preoperative severity, but remained at an intermediate level at 24 and 48 hours off stimulation. In 13 patients with GPi-DBS for primary generalized dystonia 4 patients remained at an intermediate level of symptom re-emergence after 48 hours [10]. The authors concluded that long-term DBS might have a persistent modulatory effect on the motor network. Some further impressing cases of persistent symptom relief after discontinuation of pallidal DBS in dystonia have also been reported [3,4,11,12] and are summarized in ***Table 2***.

**Table 2:** Overview on cases with dystonia and stable symptom relief after DBS discontinuation in the literature.


AUTHOR	CLINICAL CHARACTERISTICS	CLINICAL COURSE	COMMENT

Goto et al. 2004 (3)	Idiopathic cervical dystonia, bilateral GPi DBS, Male, 53y at disease onset, DBS at 54y	Improvement with DBS: TWSTRS 25 → 5 After 22m: bilateral off and stable symptom improvement >12m	Continued pharmacotherapy, short disease duration

Hebb et al. 2007 (4)	Meige syndrome, bilateral GPi DBS, Female, 48y at disease onset, sympt. progression, DBS at 58y	Improvement with DBS: GDRS 47 → 10 After 4y: bilateral off and stable symptom improvement at GDRS 20 for >12m	Sequential DBS implant (8 months between left and right electrode) and sequential turnoff

Stavrinou et al. 2012 (12)	Acquired segmental dystonia, bilateral GPi DBS, Male, 23y at disease onset, DBS at 29y	Improvement with DBS: BFM 34 → 26 After 27m: bilateral off and stable symptom improvement >6m	Young age, acquired dystonia

Cheung et al. 2013 (2)	2 cases: #1: inherited generalized dystonia (DYT1), bilateral GPi DBS Male, 11y at disease onset, DBS at 15y #2: generalized idiopathic dystonia, bilateral GPi DBS Male, 11y at disease onset, DBS at 18y	#1: Improvement with DBS: BFM 64 → 0 After 5y: Unilateral off for 3m; after 2 more weeks mild symptom recurrence #2: Improvement with DBS: BFM 66 → 1 After 19m: Unilateral off since 7m and bilaterally for 2m; after 2 more weeks symptom recurrence	Young age, short disease duration

Ruge et al. 2014 (11)	3 cases: #1: inherited generalized dystonia (DYT1), bilateral GPi DBS Male, 8y at disease onset, DBS at 9.5y #2: inherited generalized dystonia (DYT1), bilateral GPi DBS Male, 6y at disease onset, DBS at 13y #3: inherited generalized dystonia (DYT1), bilateral GPi DBS Female, 9y at disease onset, DBS at 10.5y	#1: Improvement with DBS: BFM 89 → 5.5 After 4.5y: bilateral off and stable symptom improvement at BFM 14 >14d #2: Improvement with DBS: BFM 69 → 0 After 9y: bilateral off and stable symptom improvement at BFM 13 >12m #3: Improvement with DBS: BFM 60 → 5 After 5y: bilateral off and stable symptom improvement at BFM 5.5 >14d	Young age, short disease duration

Wolf et al. 2021	Acquired generalized dystonia, bilateral Vim DBS followed by bilateral GPi DBS; Male, disease onset in childhood, DBS at 25y	Improvement with DBS: BFM 80.5 → 67 After 76m: bilateral off and stable symptom improvement >47m	Young age, acquired dystonia, switch from bilateral Vim to bilateral GPi DBS


BFM = Burke Fahn Marsden scale; TWSTRS TSS = Toronto Western Spasmodic Torticollis Rating Scale Total Severity Score; GDRS = Global Dystonia Rating Scale; y = years; m = months; d = days.

We present a unique case of sustained benefit over years in acquired generalized dystonia after cessation of longterm chronic DBS. Our patient had sustained clinical benefit as reflected by stable improvement of the BFM scores by about 15%, which is in line with the expected benefit [13]. However, persistent improvement during 4 years after discontinuation of chronic DBS, goes beyond previous observations of prolonged benefit after DBS cessation. Effects related to neuroplasticity might have led to a mild deterioration, which was rapidly resolved by IPG replacement and restart of chronic stimulation. Similarly, as in previous reports on idiopathic or inherited dystonia our patient was under chronic DBS for several years. Our observation, however, nevertheless is remarkable since DBS is clearly less effective in acquired forms of dystonia. We posit that chronic pallidal DBS can be a genuine disease-modifying treatment in single patients with dystonia with regard to its long-term effect even after prolonged discontinuation. It remains to be elucidated, which clinical or etiologic characteristics might favour such a beneficial course.
